# Metabolomics in Autoimmune Diseases: Focus on Rheumatoid Arthritis, Systemic Lupus Erythematous, and Multiple Sclerosis

**DOI:** 10.3390/metabo11120812

**Published:** 2021-11-29

**Authors:** Naeun Yoon, Ah-Kyung Jang, Yerim Seo, Byung Hwa Jung

**Affiliations:** 1 Molecular Recognition Research Center, Korea Institute of Science and Technology, Seoul 02792, Korea; youne@sm.ac.kr (N.Y.); ahkyungjang@gmail.com (A.-K.J.); rim062000@gmail.com (Y.S.); 2 College of Pharmacy, Sookmyung Women’s University, Seoul 04310, Korea; 3 Department of Pharmacy, Graduate School, Kyung Hee University, Seoul 02447, Korea; 4 KIST School, Division of Bio-Medical Science and Technology, Korea University of Science and Technology (UST), Seoul 02792, Korea

**Keywords:** metabolomics, autoimmune disease, pharmacometabolomics

## Abstract

The metabolomics approach represents the last downstream phenotype and is widely used in clinical studies and drug discovery. In this paper, we outline recent advances in the metabolomics research of autoimmune diseases (ADs) such as rheumatoid arthritis (RA), multiple sclerosis (MuS), and systemic lupus erythematosus (SLE). The newly discovered biomarkers and the metabolic mechanism studies for these ADs are described here. In addition, studies elucidating the metabolic mechanisms underlying these ADs are presented. Metabolomics has the potential to contribute to pharmacotherapy personalization; thus, we summarize the biomarker studies performed to predict the personalization of medicine and drug response.

## 1. Introduction

Metabolomics is a field of -omics technology that comprehensively studies metabolites in organisms using high-throughput analytical technology, a collection of metabolome studies. Metabolites are known to represent the last downstream of biochemical reaction, and they are widely used in clinical study and drug discovery [1]. They are produced by the host and are involved in important cellular functions, including energy production, signal transduction, and apoptosis, and can also reflect dietary and other environmental sources [2,3]. The current metabolomics study not only looks for diagnostic biomarkers but is also an attempt to discover the mechanism that will identify metabolites for treatment. The clinical application of metabolomics aims to determine the diagnostic biomarkers of disease, pathological mechanisms, and novel drug targets and therapeutic responses [4].

Autoimmune disease (AD) is a type of chronic disease closely linked to metabolic aberrations and changes [5,6]. It is a disorder that produces responses against self-antigens, owing to a breach in self-tolerance. It includes various disorders such as multiple sclerosis, rheumatoid arthritis, and lupus, afflicting 5–10% of the global population [7]. The importance of metabolomics in autoimmune disease has been raised because it can aid in understanding the molecular mechanism behind a specific phenotype of the disease [8]. The most important unmet need regarding autoimmune disease is that most treatments only alleviate symptoms by targeting inflammatory pathways and fail to recover self-immune tolerance [9]. Some autoimmune diseases are systemic (e.g., systemic lupus erythematosus (SLE)), but others target specific tissues (e.g., multiple sclerosis (MuS)) [10].

Metabolism is an important integrator of both genetic and environmental factors, and it can control immune cell differentiation under physiological and pathological conditions [11]. Recently, the regulation of immune cell metabolism to target drug discovery for immune-mediated diseases was explored [5]. Immune cells have unique characteristics that demand bioenergetic plasticity, and bioenergetics are particularly important in autoimmune diseases [12]. A new field of metabolic study in immune cells is called immunometabolism, which is thought to provide new insights into immune system regulation in pathogenesis [5]. In addition to this, metabolomics is important in precision medicine research to provide a unique metabolic fingerprint of the patient’s disease state or drug response [13].

For these reasons, metabolism studies on autoimmune disease have been studied, and their significance has been generously discussed [5,14,15,16,17]. Herein, we comprehensively summarize the trials and advances in metabolomics research on autoimmune diseases such as rheumatoid arthritis, multiple sclerosis, and erythematosus and provide insights in this paper.

## 2. Application of Metabolomics

### 2.1. Defining Metabolomics

Metabolomics is a field of ‘omics’ technology that is defined as the identification and measurement of endogenous small molecules in biochemical processes. It aims to diagnose disease by biomarkers and provide fundamental information for developing treatment. Metabolites are measured in various biological samples and provide phenotypes that differentiate between health and disease [18].

There are two main approaches to metabolomics experiments, non-targeted and targeted metabolomics. First, non-targeted metabolomic approaches involve profiling a wide range of metabolites without a prior hypothesis [19]. They focus on simultaneous detection of all accessible metabolites in a biological sample, giving an overall view of the whole metabolome [20,21]. They are usually performed to identify whole metabolites and explore biomarkers. Secondly, targeted metabolomics is a quantitative approach to measure specific metabolites. It focuses on the quantitative analysis of pre-defined metabolites in biological samples [20,21] and is usually based on specific biochemical hypotheses that focus on relevant pathways of interest. Therefore, prior information is required to develop and optimize the method of specific metabolite analysis to assess targeted metabolomics [19], which provides higher sensitivity and selectivity than non-targeted metabolomics. As previously noted, since both approaches have completely different analytical purposes, it is important to choose the appropriate approach according to the study purpose.

### 2.2. Metabolomics Workflow

**Figure 1:**
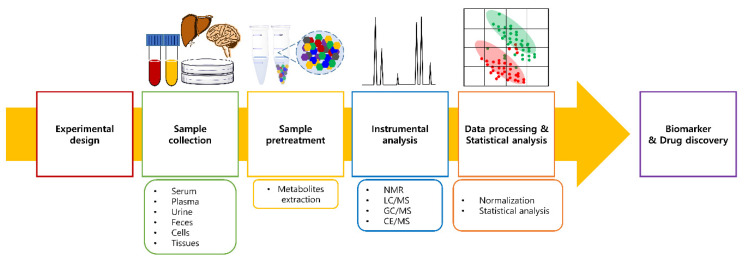
General metabolomics workflow in biomarker and drug discovery.

According to previous studies [6,18,22,23,24,25], a typical metabolomics workflow includes the following six steps: (1) experimental design, (2) sample collection, (3) sample pretreatment (metabolite extraction), (4) instrumental analysis, (5) data processing and statistical analysis, and (6) biomarker verification. A typical metabolomics workflow is shown in Figure 1.

#### 2.2.1. Sample Collection and Pretreatment

In the sample collection step, there are many types of biological samples, but the most used biological samples for biomarker discovery are serum/plasma, urine, and fecal extracts because they are minimally invasive and contain thousands of metabolites [6]. Usually, the sample pretreatment method differs according to the type of sample, metabolite of interest, and analysis platform used. For example, nuclear magnetic resonance analysis can be performed without special pretreatment of the sample, but mass spectrometry-based analysis generally requires sample pretreatment, including the extraction of metabolites [26]. Typical biological sample pretreatments for metabolomics studies include protein precipitation, extraction, and derivatization/reconstitution [27]. The most common method for protein precipitation is the use of organic solvents such as methanol and acetonitrile. Metabolite extraction is a major step in the analysis because the results of metabolomics are highly dependent on the extraction procedure. The main goals of extraction are acquiring the metabolite from the sample and removing the interfering substance [28]. The extraction method varies depending on the biological sample. Solid–liquid extraction is performed for solid samples, and liquid–liquid extraction (LLE), solid-phase extraction (SPE), or solid-phase microextraction (SPME) are applied for liquid samples [28]. LLE can separate metabolites into two parts, polar and non-polar, using organic solvents and aqueous solutions [27]. It is used to remove unwanted substances and extract the metabolites of interest. SPE uses various extraction sorbents, including reversed-phase materials and ion-exchange materials to absorb interfering substances [27]. Owing to the availability of a wide variety of sorbents, SPE allows for a more selective protocol design than LLE [27].

#### 2.2.2. Instrumental Analysis

The two main analytical platforms in metabolomics are nuclear magnetic resonance (NMR) spectroscopy and mass spectrometry (MS). NMR spectroscopy is a non-destructive technique, which means samples can be analyzed more than once [24,29]. The NMR measurement process is based on the principle that certain atoms (^1^H, ^13^C, ^31^P) within molecules can absorb characteristic radiation that occurs when the molecules are placed in very strong magnetic fields [26,30]. These strong magnetic fields change the direction of the nuclear spin in each atom in the molecule. Each molecule has a distinct pattern of NMR chemical shifts because of its chemical structure, and the arrangement of hydrogen atoms around the molecule is different [24,26]. These features allow compounds to be identified and quantified by NMR.

MS instruments detect metabolites in a completely different way than NMR instruments. MS is a destructive technique, for, in all MS-based techniques, ionization of the molecules is the key to metabolite detection and identification [24]. Compounds can be identified by measuring the mass-to-charge (*m*/*z*) ratio or fragments of the ionized molecules [24]. MS is usually combined with other separation techniques such as liquid chromatography (LC), gas chromatography (GC), and capillary electrophoresis (CE) [19].

There are three differences between NMR and MS methods. Firstly, the MS method is thousands of times more sensitive than NMR methods [6]. NMR loses information related to metabolites present in a low concentration of the sample [19]. Secondly, unlike the MS method, NMR analysis does not require special pretreatment of the sample, usually requiring only dilution of the sample [6]. Thirdly, the amount of sample used for NMR analysis is typically 300 μL, but MS-based analysis requires only 10–30 μL [6].

#### 2.2.3. Sample Normalization

Regardless of which of the two analytical platforms is used, all metabolomics analysis requires the use of internal standards (IS) and quality control (QC) samples [29,31,32]. Internal standards (IS) are known compounds at known concentrations that are added to the biological sample, and quality control (QC) represents the metabolite composition of the samples [24]. They help to perform accurate and precise metabolomics analysis of the actual concentration differences of individual metabolites found in different samples and to ensure that the run is satisfactory [26,33]. Because whole sample amounts or concentrations of metabolites can be considerably different from sample to sample, it is crucial to reduce or eliminate the effect of variation [33].

Furthermore, sample normalization is also an essential process. Sample normalization methods differ depending on the type of sample. Urine is a commonly used biofluid in metabolomics, and creatinine is the most commonly used reference. It is assumed that the creatinine concentration reflects the urine concentration [33,34]. For mammalian cells, cell counting is commonly used for cell amount normalization. For many other biofluids or biological samples, there is no known compound that is widely accepted as a reference for sample normalization, and it can vary greatly [33].

#### 2.2.4. Statistical Analysis

In large-scale metabolomic data sources, appropriate statistical analysis is essential to extract meaningful results. There are generally two types of approaches in multivariate statistics. The first approach is unsupervised learning, which identifies patterns in the data set without knowing any labels or scores [25]. Unsupervised learning finds occurring patterns autonomously and automatically groups and clusters to compress the data set and extract meaningful results [25]. In unsupervised learning, the most common technique is called principal component analysis (PCA), which is aimed at reducing the dimension of the number of metabolites analyzed when there is a significant correlation between metabolites in a given data set [24,35]. The other approach is supervised learning. Supervised learning is a method of data interpretation by knowing label information [25]. It provides an inferred function that can be used for mapping labeled data [25]. In supervised learning, the most common technique is partial least squares discriminant analysis (PLS-DA), which finds the projection direction that gives the largest covariance between the original data and the labels [24,25]. In this way, it is possible to describe the efficacy of a treatment strategy and the progression of the pathology [36].

## 3. Metabolomics in Biomarkers of ADs

### 3.1. Discovery of Biomarkers in ADs

Numerous metabolomics studies have been conducted on autoimmune diseases. One important goal of metabolomics is to develop biomarkers that can identify and diagnose disease based on changes in metabolite levels. The cause of most autoimmune diseases such as rheumatoid arthritis, multiple sclerosis, and systemic lupus erythematosus is currently unclear. It is related to genetic factors, environmental factors, gut microbiota, gender, etc. [37]. Because of these disease characteristics, it is important to systematically identify differences between healthy people and AD patients through metabolomics and to discover biomarkers for diagnosis and treatment.

#### 3.1.1. Biomarkers of Rheumatoid Arthritis (RA)

In previous studies, metabolic alterations have been reported between HCs (healthy controls) and RA (rheumatoid arthritis) patients using serum, urine, and synovial fluid. We summarized a list of altered metabolites in patients with RA in Table 1.

In a clinical study on RA patients, amino acids such as isoleucine, valine, methionine, threonine, alanine, and histidine significantly decreased. Glucose and lactic acid also changed in the RA group, showing different trends in all studies. Glucose metabolism not only provides energy for physical activity but also forms a complex network of signals that mediate various physiological functions [42]. These metabolite changes are related to glycolysis, TCA cycle, amino acids, and lipid metabolism. Amino acids were found at lower concentrations in the RA patients group. This result may indicate that proteins were degraded into amino acids in response to energy homeostasis, inflammation, and autoimmunity responses [39].

Citric acid also tends to downregulate in RA patients, indicating that the aerobic metabolic process was weakened [39]. The citric cycle is the major metabolism for all aerobic decomposition, and a downward trend of citric acid means a reduction of energy productivity under inflammatory conditions [49]. Citric acid is the most potent metabolite for the diagnosis of ADs and is thought to be highly related to disease activity [44]. Various lipid metabolites are involved in the development and progression of RA. Patients with RA with active inflammation have low total cholesterol levels [38].

#### 3.1.2. Biomarkers of Multiple Sclerosis (MuS)

Several metabolomics studies have been conducted to elucidate biomarkers of multiple sclerosis in biological fluids such as cerebrospinal fluid (CSF), plasma, urine, and brain tissue. Table 2 shows the metabolic changes in patients with MuS.

CSF is the most valuable biological sample in that it provides a deeper understanding of CNS disease because it serves as an interface between blood and brain tissue and directly provides a MuS pathology [63]. Metabolite analysis and interpretation of CSF is fundamental to understanding the mechanisms of neuroinflammation, enabling biomarker discovery and disease diagnosis, and suggesting therapeutic directions. There are many challenges, however, to obtaining CSF normal controls from healthy individuals, owing to ethical issues and the invasive nature of the matrix [64]. Unlike CSF, biological samples such as blood and urine can be obtained non-invasively and safely, and various metabolic changes can be observed. They are also involved in many pathways, such as amino acid, carbohydrate, and lipid metabolism. Still, most of these are related to general inflammatory responses and cannot be used as clinical diagnostic indicators [65]. Therefore, the study of CSF metabolomics is essential to discover biomarkers for multiple sclerosis [64].

The results of CSF metabolite profiling in patients with MuS were highly correlated with the tryptophan–kynurenine pathway. There was a general tendency for inflammation to result in decreased tryptophan, increased kynurenine or kynurenic acid, and an increased kynurenine/tryptophan ratio (or a decreased tryptophan/kynurenine ratio). Quinoline acid was almost universally upregulated and picolinic acid generally downregulated as measured. Analysis of CSF metabolites in the tryptophan–kynurenine pathway maintained their potential as inflammatory biomarkers in the early diagnosis and prognosis of neuropathology [60,61,62,66,67].

Plasma profiling of MuS patients and controls resulted in decreased levels of glucose and tryptophan and increased levels of acetoacetate, acetone, choline, and alanine. Fluctuations in these metabolites were associated with changes in the tryptophan pathway and energy metabolism and had similar results to metabolic changes in CSF [53].

#### 3.1.3. Biomarkers of Systemic Lupus Erythematosus (SLE)

As with rheumatoid arthritis, metabolic changes in biological samples from patients with SLE were related to glycolysis, TCA cycle, and amino acid metabolism. Table 3 shows the metabolic changes in patients with SLE.

Generally, in serum or plasma, most of the metabolites required for energy production associated with these pathways have been shown to decrease in SLE patients. Some studies have shown that glycolysis is inhibited in SLE. It was found that glucose increased but lactic acid decreased [70,71]. Similarly, several studies have shown that TCA cycle intermediates are decreased in SLE, suggesting decreased activity of the TCA cycle in SLE. Inhibition of glycolysis due to a decrease in the TCA cycle intermediate reflects the systemic inflammatory response rather than a response to specific end-organs because SLE is a systemic disease.

In addition, most amino acids analyzed in SLE were generally downregulated in serum [39,69,71,72], but contradictory results were found in the results of analyses of feces and urine [69,75].

Most metabolic changes were consistent with lipid metabolites in patients with SLE and have been reported to regulate immune responses and disease progression [77,78]. Arachidonic acid, a precursor of many inflammatory mediators, has been observed in several studies and tends to decrease in the serum of most SLE patients.

#### 3.1.4. Comparing Biomarkers of ADs

**Figure 2:**
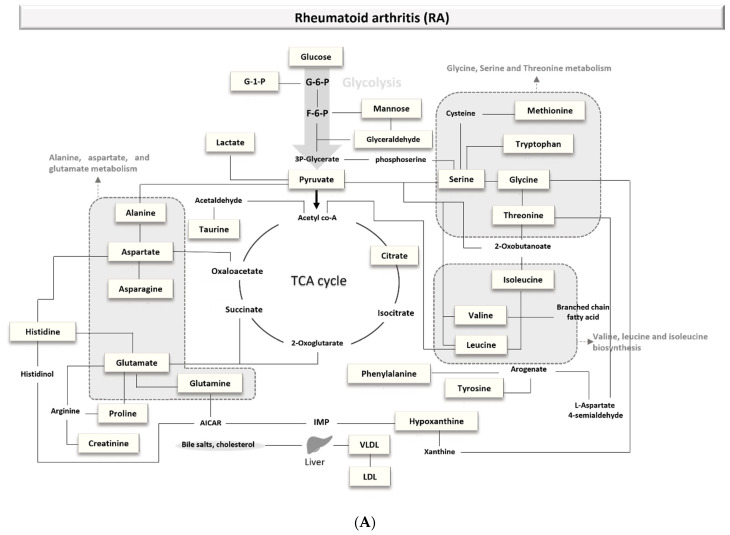

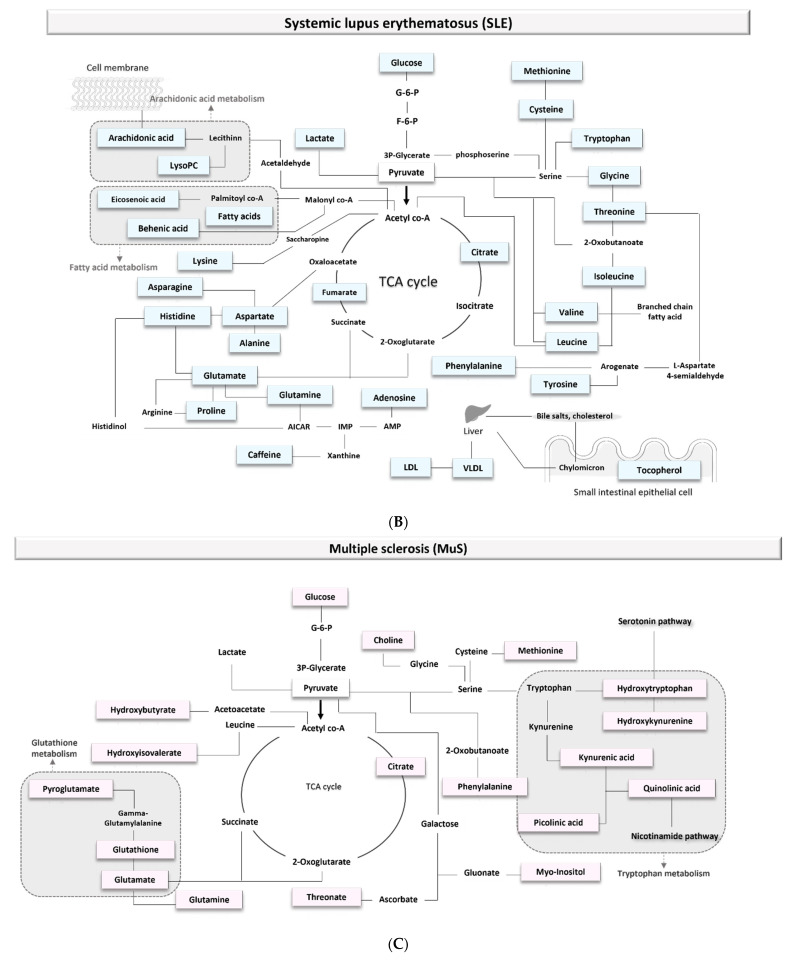
Overview of metabolic pathways involved in ADs. (**A**) Rheumatoid arthritis (RA); (**B**) Systemic lupus erythematosus (SLE); (**C**) Multiple sclerosis. TCA, tricarboxylic acid cycle or Krebs cycle; G-1-P, glucose 1-phosphate; G-6-P, glucose 6-phosphate; F-6-P, fructose-6-phosphate; AMP, adenosine monophosphate; IMP, inosine monophosphate; AICAR, 5-Aminoimidazole-4-carboxamide ribotide; LDL, low-density lipoprotein; VLDL, very-low-density lipoprotein.

A mapping network was generated by comparing the major biomarkers of the three ADs (Figure 2). Glutamate, amino acids (glycine, phenylalanine, methionine), glycolysis-related metabolites (glucose), and TCA cycle-related metabolites (citrate) are commonly altered in three ADs.

However, specific metabolisms are also changed with each disease. Amino acid metabolisms (glycine, serine, and threonine metabolism; valine, leucine, and isoleucine biosynthesis; alanine, aspartate, and glutamate metabolism) are mainly involved in RA (Figure 2A).

Arachidonic acid metabolism and fatty acids metabolism have been associated with SLE (Figure 2B). SLE is a chronic disease that causes inflammation, and arachidonic acid is a representative metabolite associated with inflammation [79]. In addition to this, fatty acids affect inflammation through various mechanisms [80].

Interestingly, the tryptophan and glutathione metabolisms change in MuS patients (Figure 2C). MuS causes inflammation and degeneration in the central nervous system. Tryptophan is utilized for the generation of several neuroactive compounds [81], such as in the synthesis of the aminergic neurotransmitter serotonin and the neurohormone melatonin [82]. Nourbakhsh et al. reported that the tryptophan metabolism of the gut microbiota and the kynurenine pathway could be relevant to the risk of MuS in children [83]. Overall, the pathway associated with these biomarkers can be outlined to characterize the disease.

### 3.2. Limitation of Current Biomarkers

Metabolomics-based studies have provided a potential means for diagnosing disease, determining disease course, predicting progression, and evaluating the effectiveness of drug responses. There are several challenges to discovering biomarkers for more accurate and personalized disease-specific diagnosis and prognosis. Despite the useful prospects of metabolomics, its limitations need to be overcome for clinical practice and research. Metabolic analysis requires the appropriate experimental method and statistical analysis to identify metabolites. For this, a metabolomics study needs expertise not only in analytical chemistry but also a combination of analytical techniques, statisticians, and biologists [84].

Another problem is that discovered metabolites are non-specific and lack diversity. Most of the identified biomarkers in metabolomics have failed to replace clinical tests, and the pathway-based approach has been suggested as a key technology in metabolomics [85]. Metabolomics cannot work alone because it is difficult to find very specific metabolites for disease. Different trends in metabolites also render biomarkers less successful.

Even in patients with the same disease, metabolic changes may show contradictory results from variables such as medications, co-morbidities, and other environmental influences. For example, a metabolic difference between races has been reported [86,87]. Small changes in physiology can also significantly influence the metabotype [1,16]. A multitude of single nucleotide polymorphisms in metabolic enzymes such as cytochromes P450 (CYPs) and uridine 5′-diphospho-glucuronosyltransferase (UGTs) can contribute to metabolic differences between individuals [88,89,90]. The expression of these enzymes affects drug metabolism, hormone synthesis, and breakdown.

These biological differences may have resulted in different trends in the metabolic profile of the same disease. Different results may also have been obtained owing to differences in the biological sample types, analytical methods, instruments, etc. The standardization of sample collection and processing methods is important in metabolomics analysis [91].

While an ideal biomarker that could diagnose a disease with a single metabolite is desirable, multiple biomarkers involved in various metabolic processes may be the best possible way of diagnosing ADs. As is commonly accepted, an integrated approach between metabolomics and other omics is needed [92].

## 4. Metabolomics in Drug Discovery for ADs

### 4.1. A New Target Discovery

Discovering the metabolic markers can help to target enzymes involved in key processes and develop cost-efficient and effective drugs for better disease treatment [93]. The metabolic imbalances that underlie ADs are poorly described, and there have been attempts to discover the metabolic mechanism underlying ADs. Herein, we introduce a metabolic mechanism study on representative ADs such as rheumatoid arthritis (RA), multiple sclerosis (MuS), and systemic lupus erythematosus (SLE) in this paper.

Recently, immunometabolism study has increased over the past decade, and it is dedicated to discovering the metabolic mechanism of immune cell function. Immune cells have their own unique metabolic characteristics. Depending on the type of immune cells, they require a different metabolic change to generate energy [94]. The metabolic rewiring of immune cells is known to be a promising target of novel drug discovery. ADs cause the metabolic rewiring of immune cells, and the metabolic aberrations in cells contribute to the inflammatory phenotype in ADs.

Activated T cells and B cells should respond to signals including synthesis cytokines, migration, and massive expansion. Therefore, the immune response requires immense energy and its biosynthetic precursors. Activated B cells that require metabolic reprogramming depend on Glut1 (Glucose transporter 1) to support proliferation and antibody production that is distinct from T cells [95]. T lymphocytes play a critical role in defense and immune response [94,96]. Glucose is an important energy source in proliferating T cells, and the triggering T-cell receptor (TCR) enhances the mitochondrial function but also increases extramitochondrial glycolysis for rapid ATP generation [97]. The metabolic characteristics of immune cells are different for each AD, just as immune cells have unique metabolic properties. Some ADs dysregulate metabolisms such as glycolysis, glutaminolysis, or the kynurenine pathway (KP), depending on each characteristic [58,98,99], and this metabolic vulnerability could be a new target for drug discovery. In this part, we briefly described the metabolic characteristics of each disease and their potential as a target.

#### 4.1.1. Rheumatoid Arthritis (RA)

Rheumatoid arthritis (RA) is an autoimmune disease characterized by persistent immune activation [100,101]. The immoderate cytokine production, dysregulated proliferation of synovial fibroblasts, formation of complex lymphoid microstructures in inflamed joints, autoantibody production, and uncontrolled activity of bone-destructive osteoclasts are pathogenic functions in RA [12]. It has been suggested that a specific metabolic alteration could be a therapeutic target in RA [99]. For example, glycolysis, glutaminolysis, choline metabolism, amino acids, and fatty acid synthesis have been proposed as therapeutic targets for RA [98,99,102,103,104,105,106,107]. Additionally, proteins involved in these pathways such as pyruvate kinase M2 (glycolysis), hypoxia-induced factor (glycolysis), GLS1 (glutaminolysis), and choline kinase (choline metabolism) have been suggested as therapeutic targets [104,107,108,109]. Targeting these metabolisms can provide a chance for disease modulation and restoration of homeostasis.

As in tumors, glucose uptake has been observed in metabolically active joints or other inflammatory sites in RA [99]. In addition, elevated glycolysis activity is associated with hypoxia in RA synovial membranes [110], and anaerobic glycolysis was related to an increasing degree of RA synovial vascularity and inflammation [111]. Aberrant glycolysis metabolism was related to RA, and the association of its markers with RA was investigated. Hypoxia-induced factor (HIF-1) is required for glycolysis induction and arouses the transcription of genes involved in glucose uptake and glycolysis [112,113]. It is a transcription factor that orchestrates adaptation to hypoxia environments [114] and regulates the angiogenesis process and glycolysis metabolism. HIF-1 increases glucose uptake and anaerobic glycolysis through the upregulation of glucose transporter 1 (GLUT1) [115]. HIF-1 was reported to be highly expressed in RA [116] but not expressed in patients with osteoarthritis (OA). HIF-1 factor was also related to the VEGF (vascular genesis) and c-myc. VEGF can induce angiogenesis in RA and FLS, a typical characteristic of RA. Since HIF-1 is associated with the alteration of glucose metabolism, changes in glucose metabolism in RA may be related to HIF-1. The genes related to the glycolytic pathway, such as HK2, LDHA, and PDK1, affected by HIF-1, play important roles in the RA fibroblast-like synoviocyte (FLS) phenotype [112]. HIF-1 has a strong association with RA and could be a therapeutic target. At this point, a tylophorine-based compound reduced inflammation in RAW2.4 cells and meliorated the severity and incidence of collagen monoclonal antibody-induced rheumatoid arthritis in a mouse model by targeting the HIF-1 and c-myc [117]. Succinate remodeled the HIF-1α/VEGF axis to induce synovial angiogenesis and suppressed succinate dehydrogenase (SDH) to prevent succinate accumulation results in inhibition of the HIF-1α/VEGF axis, showing the potential to attenuate revascularization in arthritis [118].

For decades, the infiltration of CD4 T cells in RA patients’ synovial joints has been reported [119]. Thus, the metabolic characteristics of CD4 T cells in RA patients were studied in an in vitro study [12]. Aerobic glycolysis was expected to be upregulated rapidly when naive CD4 T cells were transitioned into effector T cells by stimulating T-cell receptors, but RA T cells failed to produce as massive ATP and lactate as healthy control T cells in HLA class II-associated RA [12,120]. In the RA patients’ CD4 T cells, they favor lipogenesis rather than mitochondrial activity and have low ROS concentrations, which impose reductive stress instead of oxidative stress [97]. RA T cells also have been reported to skew toward fatty acids [121]. Retarding lipogenesis by inhibiting fatty acid synthesis can reduce tissue inflammation and correct the tissue-invasive and arthritogenic behavior of RA T cells [122]. This suggests regulating T-cell metabolism in RA as a new therapeutic target.

Contrary to this concept, omega-3 fatty acids have anti-arrhythmic action and can reduce inflammation, and they have been proposed as therapeutic agents [123,124,125,126,127]. In a clinical study, the therapeutic effects of omega-3 fatty acids were evaluated for decades. Fish oil (containing omega-3 fatty acids) ingestion decreased the production of leukotriene B4 (LTB4), tender and swollen joints, and improved physician assessments of pain and disease [127]. From these studies, omega-3 fatty acids have been shown to reduce disease and have an anti-inflammatory action in RA.

#### 4.1.2. Multiple Sclerosis

Multiple sclerosis (MuS) is a chronic inflammatory, demyelinating, and neurodegenerative disorder in the central nervous system (CNS) [128]. The immune system attacks the myelin sheath and the cells that comprise it; thus, de novo lipid synthesis for myelin remodeling and repair is critical in MuS [129]. Clinical trials have investigated statin inhibitors of HMG-CoAR and cholesterol biosynthesis as an add-on therapy for MuS patients [130]. Studies of statins in a murine MuS model have shown decreased disease severity but could not be converted to a proven effect in relapsing MuS in humans [131].

It was also discovered that the majority of alterations in MuS were related to energy metabolism [58,130]. The kynurenine pathway (KP) has a strong association with MuS, and activation of the KP results from chronic inflammation [58,132]. Some KP metabolites play a neuroprotective role (kynurenic acid, picolinic acid, and the cofactor nicotinamide adenine dinucleotide), but others play a neurotoxic role (quinolinic acid, 3-hydroxykynurenine) [132]. The KP accounts for ~95% of overall tryptophan degradation, and it is rate-limited by indoleamine 2,3-dioxygenase (IDO), which is regulated by IFN-γ and cytokines [133]. The connection between the KP and MuS was discovered in 1979, and it showed lower levels of tryptophan in MuS patients compared to controls [133]. IDO-1 expression and KYN levels were decreased in the peripheral blood monocytes (PBMCs) of relapsing–remitting MS (RRMS) patients compared to healthy controls [134]. PBMCs from MuS patients showed reduced amino acids, and this reduction decreased regulatory T cells, with an increase in myelin basic protein-specific T cell proliferation and proinflammatory cytokines secretion [134]. This study marked the importance of the KP as a promising target for the development of drugs for the treatment of MuS. Several compounds related to the KP have been developed for the treatment of MuS; endogenous tryptophan metabolites, structural analogs, IDO inhibitors, and kynurenine-3-monooxygenase inhibitors have been investigated [135,136].

In addition, sterol and bile acids have been explored in association with MuS, producing a loss of myelin oligodendrocytes in the CNS related to MuS. Zita Hubler et al. discovered that the accumulation of the 8,9-unsaturated sterol is a key mechanistic node that promotes oligodendrocyte formation through GC-MS-based profiling [137]. Recently, lower levels of multiple primary and secondary bile acids were observed in patients with MuS compared to controls [128,138]. The supplementation of tauroursodeoxycholic acid (TUDCA) has been demonstrated to reduce the severity of disease through G protein-coupled bile acid receptor 1 (GPBAR1) in experimental autoimmune encephalomyelitis (EAE) [138]. The suppression of immune cell proliferation represents a successful treatment strategy in T-cell-mediated autoimmune diseases such as RA and MuS. The inhibition of dihydroorotate dehydrogenase (DHODH) that mediates de novo pyrimidine synthesis showed repertoire diversity in patients with RRMS [139]. Klotz et al. revealed that DHODH inhibition priorly suppressed the proliferation of high-affinity T cells and suggested that increased susceptibility to DHODH inhibition resulted from high-affinity T cells, preferably using oxidative phosphorylation (OXPHOS) in the early activation stage [139]. These studies suggested that the metabolic vulnerability caused by MuS could be the therapeutic target.

#### 4.1.3. Systemic Lupus Erythematosus (SLE)

Systemic lupus erythematosus (SLE) is a chronic autoimmune disease that affects multiple organs with diverse clinical features [140]. The metabolomics studies of SLE patients’ serum/plasma mainly have reported reduced energy substrates from glycolysis, TCA cycle, fatty acid β oxidation, and glucogenic and amino acid metabolism [91,141]. SLE showed a decrease in amino acids such as arginine, which upregulates the levels of nitric oxide (NO) metabolites related to oxidative stress [70]. Increased nitro-oxidative stress can modulate the severity of the disease and play a role in the pathology of SLE [77]. Disease severity allied with oxidative stress and apoptosis is associated with glutathione (GSH) depletion [142,143]. GSH also decreased in the peripheral blood of SLE patients [144].

In SLE patients, N-acetylcysteine (NAC), a precursor of GSH, reversed the depletion of GSH, blocked mTOR1 activation, and improved disease severity in lupus [145]. It was reported that mTOR reduced the development of CD4+/CD25+/Foxp3+ regulatory T cells [146], and they are known to be deficient in patients with SLE [147]. NAC enhanced the NADPH and reduced kynurenine in SLE patients in vivo, and kynurenine accumulation potentially contributed to mTOR activation and may be a therapeutic target [148].

Th1, Th17, regulatory T (Treg) cells, and CD4-/CD8- T cells are involved in the development of organ inflammation in SLE through distinct mechanisms [134,149,150]. Because glutaminolysis plays a key role in the generation of pro-inflammatory effector T cells, Th1 and Th17 cells, the enzymes involved in glutaminolysis have been explored. Glutaminase, which converts glutamine to glutamate, promotes Th17 cells through distinct mechanisms. Bis-2-(5-phenylacetamido-1,3,4-thiadiazol-2-yl)ethyl sulfide (BPTES), which are glutaminase inhibitors, reduced Th17 cell differentiation and disease action in an EAE animal model [151]. Like other autoimmune diseases, Lupus CD4^+^ T cells of SLE patients showed a high level of glucose metabolism [152,153]. Michihito Kono et al. revealed that BPT ES affects glycolysis as well as glutaminolysis by decreasing Hif1α protein in Th17 cells [154].

### 4.2. Metabolomics Applications in Precision Medicine

Precision medicine comprises tailored therapies for each individual and contributes effective drug treatment while avoiding the off-target effect of drugs [155]. Predicting treatment response is a useful tool to bring us one step closer to precision medicine. This is necessary for strategies for future medicine. The likelihood of a response will be known before exposure to a drug, and we can avoid drugs with little potential for efficacy, saving time, minimizing costs, and improving risk/benefit ratios [156]. The effect of drugs involves many different enzymes, multiple organs, and the microbiome [157]. As previously reported, current pharmaceutical treatments have no effect for 30–60% of patients [158,159]. Patients have different clinical characteristics that affect the drug metabolism, which results in a different drug response even if the same drug is taken. To understand the individual characteristics, clinical pharmacology can benefit from metabolomics technology.

The metabolome is a comprehensive and most informative level that provides an overview of the physiologic status [159]. Pharmacometabolomics is a branch of metabolomics that has the potential to contribute to pharmacotherapy personalization [160]. The effects of drugs on individuals and the factors that alter drug metabolism can be detected using pharmacometabolomics. Above all, it can identify biomarkers that are related to the patient’s response to drug administration [159]. In Table 4, we summarize the biomarker studies that predicted drug response in AD patients.

#### 4.2.1. Rheumatoid Arthritis (RA)

Treatment for RA has recently been well-developed. RA patients receive disease-modifying antirheumatic drugs (DMARDs) that can reduce the symptoms and signs of the disease [178]. Methotrexate (MTX) has been used in the treatment of RA and is often the first-line medication for RA treatment [179]. Up to 50% of patients, however, do not achieve a clinically adequate outcome when treated with MTX. TNF inhibitors such as etanercept (ETA), infliximab, and adalimumab are also widely used biologic agents in RA because TNF-α is a pro-inflammatory cytokine that is important for regulating the inflammatory response in RA. However, 30–40% of the patients undergoing biological treatment showed an ineffective response [168].

Metabolomics has been a useful tool for predicting patient responses to these treatments in RA [45,161,162,163,164,166,167,168,169,170,171]. These studies have investigated biomarkers to predict clinical response to the therapies and were mainly conducted with MTX or TNF inhibitor treatment. 1H-NMR, acetate, aspartate, histidine, tryptophan, hypoxanthine, and uric acid were commonly discovered as biomarkers for the response to MTX [161,162]. The urine metabolomes of 16 RA patients were screened, and histamine, glutamine, xanthurenic acid, and ethanolamine were suggested as markers predicting anti-TNF agent response with a sensitivity of 88.9% and a specificity of 85.7% [163]. Artacho et al. reported that significant associations of the gut microbiome and their genes with future clinical response, including orthologs related to purine and MTX metabolism [169]. In the BiOCURA (Biologicals and Outcome Compared and predicted Utrecht region in Rheumatoid Arthritis) cohort of 105 RA patients taking TNF antagonists, combining four metabolites with several clinical parameters correctly classified 60% of patients by responder status. The selected predictors were sn1-LPC(18:3-ω3/ω6), sn1-LPC(15:0), ethanolamine, and lysine [165].

However, Mateusz Maciejewski et al. showed that serum lipid levels during pre-treatment or early treatment are unfit for classifying the response to MTX in the routine clinical care setting [170]. Since this study was conducted for four weeks after drug administration in RA patients treated with MTX, the short study period was considered unsuitable.

#### 4.2.2. Multiple Sclerosis (MuS)

Research to discover biomarkers for the response after treatment in multiple sclerosis has been relatively recent. The first disease-modifying treatment available to treat MuS was interferon beta (IFNβ); four IFNβ agents are approved to treat MuS [180]. IFNβ responder and non-responder patients have different levels of lactate, acetone, 3-OH-butyrate, tryptophan, citrate, lysine, and glucose [180]. Glatiramer acetate (GA) is also an immunomodulating agent for the treatment of MuS; the predictive role of PTX-3 protein and metabolites (lactate, tyrosine, hypoxanthine, hydroxyproline, ADP, citrulline, ornithine, and tryptophan) was revealed by analyzing plasma from patients [175].

Neutralizing anti-drug antibodies (ADAs) can greatly reduce the efficacy of treatment for MuS, depending upon ADA-positive (ADA+) or ADA-negative (ADA–) status during the first year of treatment. Between ADA+ and ADA–, significantly different metabolites were found in the serum of patients, mostly in the lipids; M-HDL-TG or XXL-VLDL-FC were observed [174].

It was demonstrated that frankincense extract reduces disease activity in RRMS without toxicity [181,182]; its therapeutic response was investigated by metabolomics, and different levels of 12- and 15-lipoxygenase products were identified [173].

#### 4.2.3. Systemic Lupus Erythematosus (SLE)

The research on therapeutic response in lupus using metabolomics has not yet been well studied. We were able to find a metabolomics study in which lupus nephritis patients were treated with cyclophosphamide. Cyclophosphamide is a general treatment for severe organ-threatening SLE [183]. These studies can reveal altered metabolites after drug treatment but cannot show the difference between a drug responder and a non-responder [176,177]. Citrate levels were changed in the urine sample, and lipid metabolites and acetate were changed in the serum sample. In the serum of lupus nephritis (LN) patients, lipid metabolites increased but acetate decreased after six months of treatment [176]. Decreased citrate levels in the urine of LN patients improved after six months of cyclophosphamide treatment, which has been proposed as a non-invasive biomarker for monitoring treatment response in LN [177].

## 5. Conclusions

Metabolomics is a powerful tool that can discover biomarkers and provide new insight into autoimmune diseases. Within a decade, in-depth research has become possible, and much has been studied with the development of high-throughput technology. In particular, the collaboration of molecular biology and metabolomics can reveal more precise disease mechanisms. Autoimmune diseases can be studied at the cellular level with the introduction of immunometabolism, enabling a deep understanding of pathogenesis and new drug targets. Attempts are being made to alleviate diseases by blocking metabolic mechanisms such as glycolysis and glutaminolysis, which have been revealed through these studies.

Despite the advantages of the metabolomics approach, limitations remain. Many studies have identified the biomarkers from metabolomics studies but lack disease-specific properties. As autoimmune disease is a disorder related to inflammation, its characteristics are difficult to distinguish from those of inflammation or cancer.

To make up for specificity, determination can be supplemented by multi-omics studies with genes or proteins. Recently, combining different omics technologies (such as genomics, and proteomics) has been suggested to overcome and compensate for the shortcomings of metabolomics. Metabolomics has the potential to complement genomics studies, which often lack functional information on the biological process [184,185]. The genetic or proteomic data can contribute to metabolomic studies reaching their full potential [186].

Combining -omics fields can address the interaction between environmental influences and biological information and thus can provide a critical biomarker and pathological understanding. Furthermore, multi-omics reinforces the reliability of metabolomics studies. Xiaojing Chu et al. revealed that arachidonic acid pathways have a significant impact on cytokine production, and the rs174584-FADS2 locus is related to arachidonic metabolism by the integration of metabolomics and genomics study [187]. It was also reported that combined proteome and metabolome data provide efficient and reliable discrimination between healthy and diseased rats at the onset of EAE [188].

The integration of data from different ‘omics’ platforms can provide multidimensional insight into this relationship. However, only a few studies have investigated combined -omics approaches, and the right standard of -omics data for integration still needs to be further improved. Moreover, we observed that fewer studies researched SLE and MuS using metabolomics. Further metabolic study of precision medicine in these fields should be carried out. Overall, we look forward to further developments in metabolomics combined with other studies.

## Figures and Tables

**Table 1:** Metabolic changes and related studies in patients with rheumatoid arthritis (RA).

Date	Sample	Instruments	Upregulated	Downregulated	Ref.
2011	Plasma	GC-MS LC-MS	Glyceric acid, D-ribofuranose, Hypoxanthine	Histidine, threonic acid, methionine, cholesterol, asparagine, threonine	[38]
2011	Serum	^1^H NMR	Glucose, glycoprotein, lactate, VLDL, LDL	Valine, tyrosine, pyruvate, lysine, phenylalanine, HDL, cholesterol, isoleucine, histidine, alanine, phosphocholine, glycerol, glutamine, glutamate, creatinine, citrate	[39]
2009	Serum	^1^H NMR	3-hydroxybutyrate, lactate, acetylglycine, taurine, glucose	LDL, alanine, methylguanidine	[40]
2013	Serum	GC/QTOF-MS LC/QTOF-MS	Lactic acid, dihydroxyfumaric acid, glyceraldehyde, aspartic acid, homoserine	4,8-dimethylnonanoyl carnitine	[41]
2015	Synovial fluid	GC/TOF-MS	Lactic acid, carnitine, diglycerol, pipecolinic acid beta-mannosylglycerate,	Valine, citric acid, gluconic lactone, glucose, glucose-1-phosphate, mannose, 5-methoxytryptamine, D-glucose, ribitol	[42]
2016	Serum	GC-MS	Docosahexaenoate, palmitelaidate, oleate, trans-9-octadecenoate, D-mannose, glycerol, ribose	2-Ketoisocaproate, isoleucine, leucine, serine, phenylalanine, pyroglutamate, methionine, proline, threonine, valine, urate	[43]
2016	Urine	^1^H NMR	Tyrosine	N-acetyl amino acids, citrate, alanine	[44]
2016	Serum	^1^H NMR	3-hydroxyisobutyrate, acetate, NAC, acetoacetate, acetone	Isoleucine, lactate, alanine, creatinine, valine, histidine	[45]
2018	Serum	LC-MS	4-methoxyphenylacetic acid, glutamic acid, L-leucine, L-phenylalanine, L-tryptophan, L-proline, glyceraldehyde, fumaric acid, cholesterol	Capric acid, argininosuccinic acid, bilirubin	[46]
2019	Serum	LC-MS	Glutamine	Taurine, asparagine, serine, glycine, ethanolamine, aspartic acid, proline, threonine, sarcosine, alanine, valine, histidine, arginine, leucine, ornithine, methionine, tryptophan, phenylalanine	[47]
2021	Plasma	GC-MS		L-cysteine, citric acid, L-glutamine	[48]

**Table 2:** Metabolic changes and related studies in patients with multiple sclerosis (MuS).

Date	Sample	Instruments	Group	Upregulated	Downregulated	Ref.
2014	Serum	^1^H NMR	MuS	Lysine	L-Glutamine, valine	[50]
2014	CSF	^1^H NMR	MuS	Threonate, choline, myo-inositol	Phenylalanine, mannose, citrate, 3-hydroxybutyrate, 2-hydroxyisovalerate	[51]
2015	CSF	MALDI-TOF-MS, LC-MS/MS	MuS	L-glutamate		[52]
2016	Serum	^1^H NMR	MuS	Alanine, acetoacetate, acetone, choline, 3-hydroxybutyrate	Tryptophan, 5-hydroxytryptophan, glycerol, glucose	[53]
2016	CSF	GC/MS	MuS	1-Monopalmitin, 1-monostearin, pentadecanoic acid, oleic acid, methionine, valine, phenylalanine, tyrosine, leucine, proline, threose, isoleucine, putrescine, oxoproline,		[54]
2016	Urine	^1^H NMR	MuS	Trimethylamine N-oxide, 3-hydroxyisovalerate, hippurate, malonate	Creatinine, 3-hydroxybutyrate, methylmalonate	[55]
2017	Plasma	GC-MS	MuS	L-asparagine, L-ornithine, L-glutamate, L-glutamine	Pyroglutamate, fructose, myo-inositol, threonate, phosphate	[56]
2017	CSF	NMR	MuS	Pyroglutamate, 2-hydroxybutyrate, formate	Glucose, acetate, citrate	[57]
2017	CSF	UHPLC-FLD, GC/MS	MuS		L-glutamine, lactate	[58]
	Serum		RRMS	Kynurenic acid, picolinic acid		
			PPMS	3-hydroxykynurenine, quinolinic acid	Kynurenic acid, picolinic acid	
			SPMS	3-hydroxykynurenine, quinolinic acid	Kynurenic acid, picolinic acid	
2017	Serum	HPLC-ECD	SPMS, RRMS		Methionine, glutathione	[59]
2019	CSF	UPLC-HRMS	SPMS	Trigonelline, citrulline, O-Succinyl-homoserine, N6-(delta2-isopentenyl)-adenine, pipecolate, 1-methyladenosine, 4-acetamidobutanoate, 5-hydroxytryptophan, kynurenate N-acetylserotonin	3-methoxytyramine, caffeine	[60]
2020	CSF	LC-MS/MS	MuS	Kynurenine, quinolinic acid, neopterin, kynurenic acid	tryptophan, 5-hydroxy-indolacetic acid, piconilic acid	[61]
2020	CSF	LC-MS	MuS	3-hydroxykynurenine, quinolinic acid	L-kynurenine, picolinic acid	[62]
	Serum		MuS	quinolinic acid	5-hydroxyindoleacetic acid	

PPMS, primary progressive multiple sclerosis; RRMS, Relapsing-remitting multiple sclerosis; SPMS, secondary-progressive multiple sclerosis.

**Table 3:** Metabolic changes and related studies on patients with systemic lupus erythematosus (SLE).

Date	Sample	Instruments	Upregulated	Downregulated	Ref.
2011	Serum	^1^H NMR	N-acetyl glycoprotein, VLDL, LDL	Valine, tyrosine, phenylalanine, lysine, isoleucine, histidine, glutamine, alanine, citrate, creatinine, creatine, pyruvate, HDL, cholesterol, glycerol, formate	[39]
2016	Serum	GC-MS	Methionine, glutamate, cystine, 1-monopalmitin, 1-monolinolein, 1-monoolein, 2-hydroxyisobutyrate	Tryptophan, alanine, proline, glycine, serine, threonine, aspartate, glutamine, asparagine, lysine, histidine, tyrosine, valine, leucine, isoleucine, fumarate, threonate, 2-hydroxyisovalerate, carbohydrates, 2-keto-3-methylvalerate, 2-ketoisocaproate, fatty acids, aminomalonate, alpha-tocopherol	[68]
2016	Urine	GC-MS	Valine, leucine, fumarate, malate, cystine, pyroglutamate, cysteine, tryptophan, threonate, uracil, urate, pseudouridine, xanthine, glyceric acid, myo-inositol, p-cresol, glutarate, hydroxyisobutyrate, dihydroxybutyrate, 3,4,5-trihydroxypentanoic acid		[69]
2016	Serum	GC-MS	Urea, cystine, threonine, naproxen, glucose	Lysine, fumaric acid, malic acid, methionine, tyrosine, alanine, cysteine, tryptophan asparagine, threonic acid, histidine, citric acid, lactic acid, caffeine, theobromine	[70]
2016	Serum	^1^H NMR	Acetate, NAG, glucose	Leucine, valine, alanine, glutamate, citrate, choline, proline, glycine, lactate, LDL, VLDL	[71]
2017	Plasma	GC-MS	Myristic acids, palmitoleic acids, oleic acids, eicosenoic acids	Caproic acid, caprylic acid, linoleic acid, stearic acid, arachidonic acid, eicosanoic acid, behenic acid, lignoceric acid, hexacosanoic acid	[72]
2019	Feces	LC-MS	Proline, L-tyrosine, L-methionine, L-asparagine, Dl-pipecolinic acid, glycyl-L proline, L-carnosine, xanthurenic acid, kynurenic acid, 1,2-dioleoyl-rac-glycerol, lysoPE 16:0, lysoPC 22:5, PG 27:2, MG 22:6, MG 16:5	D-Ala-D-ala, lauryl diethanolamide, SQDG 26:5, adenosine, mucic acid, adenosine 5′-diphosphate, trigonelline thiamine pyrophosphate	[73]
2019	Serum	LC-MS	Ceramide, trimethylamine n-oxide, xanthine	Acylcarnitine, caffeine, hydrocortisone, itaconic acid, serotonin	[74]
2020	Feces	GC-MS	Triethylene glycol, erucamide, leucic acid, 1-phenyl-1,2-ethanediol, pyrimidine, 4-aminobutanoic acid, vaccenic acid, L-valine, L-ornithine, L-phenylalanine, L-leucine, lactic acid, arachidic acid, behenic acid, putrescine, benzoic acid, erucic acid, n-(4-aminobutyl) acetamide	2,4-di-tert-butylphenol, phosphoric acid, Glyceric acid, (Z)-13-octadecenoic acid, γ-tocopherol	[75]
2021	Serum	LC-MS	MG 20:2, L-pyroglutamic acid	Arachidonic acid, adenosine, SM 24:1, MG 17:0, lysoPE 18:0, lysoPE 16:0, lysoPC 20:0, lysoPC 18:0	[76]

**Table 4:** Relevant potential biomarkers for AD treatment outcome prediction identified through a metabolomics approach.

Disease	Year	Treatment	Sample	Instruments	Biomarker	Ref.
RA	2012	MTX	Serum	^1^H-NMR	α-oxoglutarate, glycine, citrate, aspartate, acetate, alanine, cholesterol, cysteine, histidine, hypoxanthine, lactate, glutamine, methionine, serine, taurine, tryptophan, trimethylamine-N-oxide, uracil, uric acid	[161]
	2012	Anti-TNF	Urine	^1^H-NMR	Uric acid, taurine, histidine, methionine, glycine, uracil, acetate, α-oxoglutarate, aspartate, tryptophan, hypoxanthine, TMAO, methionine, acetate	[162]
	2013	Infliximab or ETA	Urine	NMR	Histamine, glutamine, xanthurenic acid, ethanolamine	[163]
	2015	ETA	Serum	^1^H-NMR	Isoleucine, leucine, valine, alanine, glutamine, tyrosine, glucose	[164]
	2016	5 TNFis	Serum	LC-MS	Sn1-LPC(18:3-ω3/ω6), sn1-LPC(15:0), ethanolamine, lysine	[165]
	2016	Anti-TNF	Plasma	TOF-MS	D-glucose, D-fructose, sucrose, maltos	[166]
	2016	Glucocorticoids	Serum	LC-MS	Lysophospholipids	[167]
	2020	TNFis or ABT	Serum	CE-TOF-MS	Glycerol 3-phosphate, betonicine, N-Acetylalanine, hexanoic acid, taurine (TNFis) 3-Aminobutyric acid, citric acid, quinic acid (ABT)	[168]
	2020	MTX	Fecal	NMR, LC-MS	Bacteria-produced metabolites	[169]
	2021	MTX	Serum	UPLC–MS	no effect (lipidomics)	[170]
	2021	DMARDs	Plasma	NMR/MS	N-acetylgalactosamine, N-acetylneuraminic acid	[171]
MuS	2019	IFN ß	Plasma	NMR	Lactate, acetone, 3-OH-butyrate, tryptophan, citrate, lysine, glucose	[172]
	2020	SFE	Plasma	MRI	12- and 15-lipoxygenase products	[173]
	2020	IFNβ formulations	Serum	NMR	29 metabolites (e.g., TG, XL-VLDL-PL, etc.)	[174]
	2020	Glatiramer acetate	Serum	^1^H-NMR	Lactate, tyrosine, hypoxanthine, hydroxyproline, ADP, citrulline, ornithine, tryptophan	[175]
SLE	2018	Cyclophosphamide + prednisolone	Serum	NMR	Lipid metabolites and acetate	[176]
	2020	Cyclophosphamide	Urine	NMR	Citrate	[177]
